# Attenuated BK channel function promotes overactive bladder in a rat model of obesity

**DOI:** 10.18632/aging.102182

**Published:** 2019-08-21

**Authors:** Ning Li, Honglin Ding, Peng Zhang, Zizheng Li, Yili Liu, Ping Wang

**Affiliations:** 1 Department of Urology, Fourth Affiliated Hospital, China Medical University, Shenyang, Liaoning, China; 2 Department of Urology, Affiliated Hospital, Chifeng University, Chifeng, Neimeng, China; 3 Department of General Surgery, Shenyang 242 Hospital, Shenyang, Liaoning, China

**Keywords:** BK channel, overactive bladder, detrusor smooth muscle, obesity, electrophysiology

## Abstract

Overactive bladder (OAB) is mostly observed in obese individuals, and is associated with enhanced excitability and contractility of the detrusor smooth muscle (DSM). Large-conductance voltage- and Ca^2+^-activated K^+^ (BK) channels reduce the excitability and contractility of the DSM. We tested whether obesity-induced OAB is associated with altered BK channel expression and activity in the DSM. Seven-week-old female Sprague-Dawley rats (N=80) were fed a normal or high-fat diet (HFD) for 12 weeks. HFD-fed rats exhibited a higher average bodyweight and urodynamically established detrusor overactivity. mRNA levels of the *Kcnma1* (BKα subunit) and *Kcnmb1* (BKβ1 subunit) in whole tissues and cells from the DSM were reduced in HFD-fed rats. A selective BK channel opener, NS1619, was then applied to DSM cells from the two groups of rats. Patch-clamp techniques revealed that spontaneous transient outward currents, NS1619-induced activation of spontaneous transient outward currents, and whole-cell BK currents, as well as NS1619-induced membrane hyperpolarization, were attenuated in DSM cells from HFD-fed rats. The relaxation effect of NS1619 on contractility was reduced in DSM strips from HFD-fed rats. Thus, impaired expression of *Kcnma1* and *Kcnmb1* in the DSM contributes to obesity-induced OAB, suggesting that BK channels could be a useful treatment targets in OAB.

## INTRODUCTION

Overactive bladder (OAB) is characterized by urinary urgency, with or without urinary incontinence, and is usually associated with urodynamically demonstrable detrusor overactivity [1]. OAB, which affects approximately 16% of American adults, is increasing in incidence in the aging population, and significantly reduces the quality of life [2]. However, its etiology and contributing factors remain unclear.

Obesity, which is defined as abnormal or excessive fat accumulation, has been established as a common risk factor for OAB [3–7]. Data from epidemiological studies indicate that weight gain and central adiposity in adulthood are associated with a higher prevalence of lower-urinary-tract symptoms, especially OAB [8, 9]. Rats fed a high-fat diet (HFD) were found to become overweight, to develop high blood glucose and insulin concentrations, and to exhibit urodynamically demonstrable non-voiding contractions suggestive of OAB [7, 10, 11]. Obesity is associated with systemic low-grade inflammation, which may contribute to bladder dysfunction (including OAB) by increasing the concentrations of inflammatory factors in the detrusor smooth muscle (DSM) [7, 10, 11]. Moreover, OAB is associated with increased DSM excitability, contractility and spontaneous phasic contractions. Therefore, changes in the expression and function of DSM regulatory proteins may alter the contractility of the DSM [12–14].

The membrane potential constitutively controls cell excitability [15–17]. In the DSM, Ca^2+^-activated K^+^ channels are critical regulators of the membrane potential, the repolarization and afterhyperpolarization phases of the action potential, and contractility [18]. The Ca^2+^-activated K^+^ channels in the DSM are typically divided into three groups according to their biophysical properties and single-channel conductance: large-conductance voltage- and Ca^2+^-activated K^+^ (BK) channels, small-conductance Ca^2+^-activated K^+^ channels and intermediate-conductance Ca^2+^-activated K^+^ channels [18]. Small- and intermediate-conductance Ca^2+^-activated K^+^ channels are activated by Ca^2+^ but are insensitive to voltage. BK channels, which are located in the plasma membrane, are highly expressed in the DSM, and are the most important regulators of DSM function among the Ca^2+^-activated K^+^ channel family members [18–21]. Enhanced BK channel activity induces spontaneous transient outward currents (STOCs), which hyperpolarize the membrane and reduce the influx of Ca^2+^ by attenuating the activity of voltage-dependent Ca^2+^ channels. These effects ultimately relax the DSM [20–22].

BK channel conductance ranges from 120 to 240 pS in the DSM of rodents and humans [18, 19]. The BK channels consist of pore-forming α-subunits (translated from *Kcnma1*), along with four tissue-specific regulatory subunits (BKβ1-4), which are translated from *Kcnmb1-4*, with BKβ1 being specific to smooth muscle and BKβ4 being specific to neurons [15, 23, 24]. Although BKβ1 is the primary regulatory subunit in the DSM, BKβ4 mRNA and protein expression were recently detected in the DSM of rodents and humans, while the BKβ2 and BKβ3 subunits were not detected [18, 19, 23, 24]. Pharmacological inhibition or genetic deletion of the BK channels was found to increase the excitability and contractility of the DSM [23, 25, 26]. On the other hand, selective BK channel openers were reported to enhance whole-cell BK currents, thereby hyperpolarizing the DSM cell membrane potential and relaxing the DSM [23, 25, 26].

The involvement of BK channels in the etiology of OAB was recently established in patients with benign prostatic hyperplasia and neurogenic bladder dysfunction [27, 28]. In addition, BK channel expression and function were found to be attenuated in vascular smooth muscle from a HFD-fed animal model [29, 30]. However, it remains unknown whether BK channels regulate the DSM under the pathological condition of obesity.

In the present study, we used a rat model of HFD-induced obesity and a selective BK channel opener to investigate the molecular and functional changes of BK channels in the DSM. We demonstrated that reduced BK channel expression and activity contribute to obesity-associated OAB.

## RESULTS

### Changes in bodyweight

**Figure 1:**
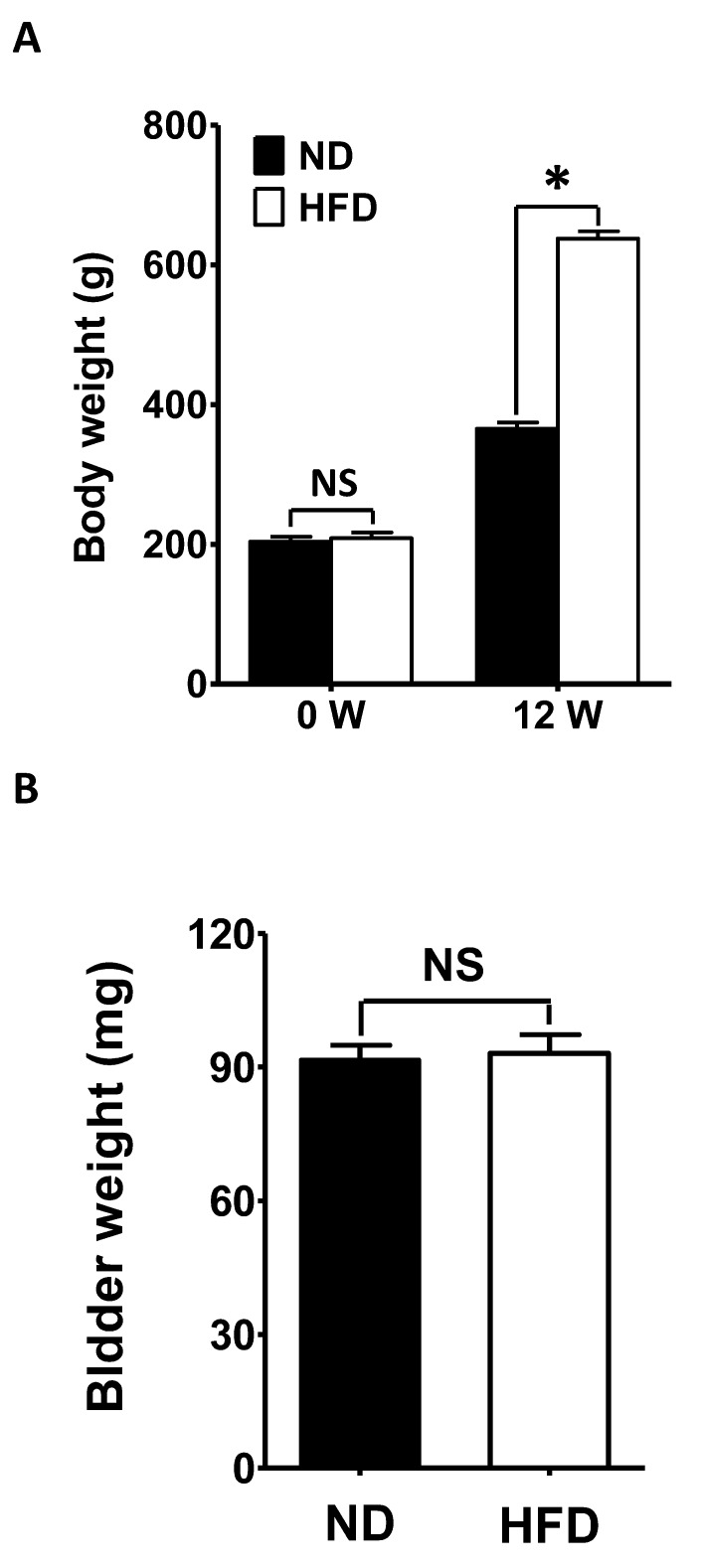
**Changes in bodyweight.** (**A**) The bodyweights before and after 12 weeks of feeding were compared between HFD rats and ND rats. (**B**) The bladder weights were compared between ND and HFD rats after 12 weeks of feeding. Data are expressed as the mean ± SEM, N = 40 per group, * P < 0.05 for ND vs. HFD. ND: normal diet; HFD: high-fat diet; W: week; *NS*: not significant.

The average weight before feeding did not differ significantly between rats fed a HFD or a normal diet (ND) (HFD 203.81 ± 7.22 g, N = 40; ND 208.72 ± 8.31 g, N = 40; P > 0.05; Figure 1A). However, after 12 weeks, the average bodyweight of the HFD rats (637.83 ± 10.51 g, N = 40) was significantly greater than that of the ND animals (365.29 ± 9.17 g, N = 40; P < 0.05; Figure 1A). On the other hand, the mean bladder weight did not differ significantly between the groups after 12 weeks (ND 91.71 ± 3.23 mg, N = 40; HFD 93.09 ± 4.24 mg, N = 40; P > 0.05; Figure 1B).

### HFD treatment successfully induces detrusor overactivity

**Figure 2:**
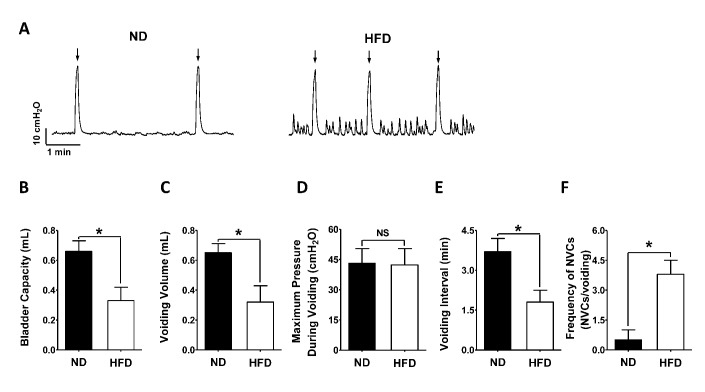
**HFD feeding successfully induces detrusor overactivity.** (**A**) Representative cystometrogram illustrating the changes in bladder function in HFD rats. A lower bladder capacity (**B**), smaller voiding volume (**C**), shorter voiding interval (**E**) and higher frequency of non-voiding contractions (**F**) were recorded in HFD rats than in ND animals. However, the maximum pressure during voiding did not differ significantly between the groups. Data are expressed as the mean ± SEM, N = 10 per group, * P < 0.05 for ND vs. HFD. ND: normal diet; HFD: high-fat diet; *NS*: not significant; NVCs: non-voiding contractions. *Arrow* indicates the voiding peak.

HFD-induced changes in urodynamic parameters of the rat urinary bladder were measured by cystometry (Figure 2A–2F). Twelve weeks after the initiation of HFD feeding, HFD rats exhibited a lower bladder capacity than ND rats (HFD 0.33 ± 0.09 mL, N = 10; ND 0.66 ± 0.07 N = 10; P < 0.05; Figure 2A, 2B), with a significant reduction in voiding volume (HFD 0.32 ± 0.11 mL, N = 10; ND 0.65 ± 0.06 mL, N = 10; P < 0.05; Figure 2A, 2C). The maximum pressure during voiding did not differ significantly between the ND (43.18 ± 7.21 cm H_2_O, N = 10) and HFD (42.31 ± 8.13 cm H_2_O, N = 10; P > 0.05; Figure 2A, 2D) rats. However, in HFD rats, the voiding interval was significantly shorter (HFD 1.83 ± 0.45 min, N = 10; ND 3.73 ± 0.47 min, N = 10; P < 0.05; Figure 2A, 2E) and non-voiding contractions were more frequent than in ND rats (HFD 3.79 ± 0.67, N = 10; ND 0.51 ± 0.47, N = 10; P < 0.05; Figure 2A, 2F), indicating that detrusor overactivity had been induced.

### The mRNA levels of the *Kcnma1* and *Kcnmb1* are significantly lower in the DSM of HFD rats

According to previous studies, the mRNA and protein expression of BKα, BKβ1 and BKβ4 subunits are measured in the DSM from human and rodents, while the BKβ2 and BKβ3 subunits are not detectable [15, 23, 24]. Therefore, we performed quantitative real-time polymerase chain reaction (qRT-PCR) to evaluate the mRNA levels of the *Kcnma1, Kcnmb1 and Kcnmb4* in DSM layers from ND and HFD rats. However, the presence of neurons, vascular myocytes, endothelial cells and fibroblasts in the DSM layer may lead to the detection of subunits that are expressed in cell types other than DSM cells. Thus, we also performed single-cell qRT-PCR on freshly isolated DSM cells to eliminate any contamination from other cell types [14, 23].

**Figure 3:**
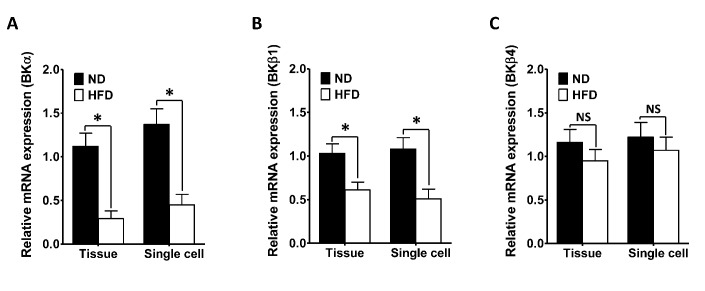
**The mRNA levels of the *Kcnma1* and *Kcnmb1* are significantly reduced in the DSM of HFD rats.** The relative mRNA levels of *Kcnma1* (**A**), *Kcnmb1* (**B**) and *Kcnmb4* (**C**) in DSM layers (Tissue) and isolated DSM cells (Single cell) were lower in the HFD group than in the ND group. Data are expressed as the mean ± SEM. Tissue: n = 16, N = 5 per group; Single cell: n = 16 (the number of strips to isolate cells), N = 5 per group. * P < 0.05 for ND vs. HFD. ND: normal diet; HFD: high-fat diet; *NS*: not significant.

Our results demonstrated that *Kcnma1 and Kcnmb1* mRNA levels in both DSM layers and cells were significantly lower in HFD rats than in ND animals (Figure 3A, 3B). There was a 3.86-fold reduction in the relative mRNA expression of the *Kcnma1* and a1.68-fold decrease in the relative mRNA expression of the *Kcnmb1* in the DSM layers of HFD rats (n = 16, N = 5) compared with those of ND rats (P < 0.05; Figure 3A, 3B). The mRNA levels of the *Kcnma1 and Kcnmb1* in DSM cells from HFD rats were 3.04-fold and 2.12-fold lower than those in DSM cells from ND rats, respectively (P < 0.05; Figure 3A, 3B). However, there was no significant difference in the *Kcnmb4* mRNA levels of the DSM layers (1.12-fold decrease, HFD vs. ND; P > 0.05; Figure 3C) or DSM cells (1.14-fold decrease, HFD vs. ND; P > 0.05; Figure 3C) of the two groups. Thus, there was a significant decrease in the mRNA expression of the *Kcnma1 and Kcnmb1*, but not the *Kcnmb4*, in whole DSM tissues and DSM cells isolated from the DSM tissues of HFD-fed rats.

### STOC activity is attenuated in freshly isolated DSM cells from HFD rats

The activation of BK channels can induce STOCs, which contribute to the hyperpolarization of the DSM cell membrane potential and prevent the firing of spontaneous action potentials that cause phasic contractions [17, 20, 21]. We used a perforated patch clamp to record STOCs from the DSM at membrane potentials of 0 mV and −40 mV. The average DSM cell capacitance did not differ significantly between the ND (27.31 ± 1.22 pF; n = 21, N = 12) and HFD (26.62 ± 1.52 pF; n = 21, N = 11; P > 0.05) groups.

**Figure 4:**
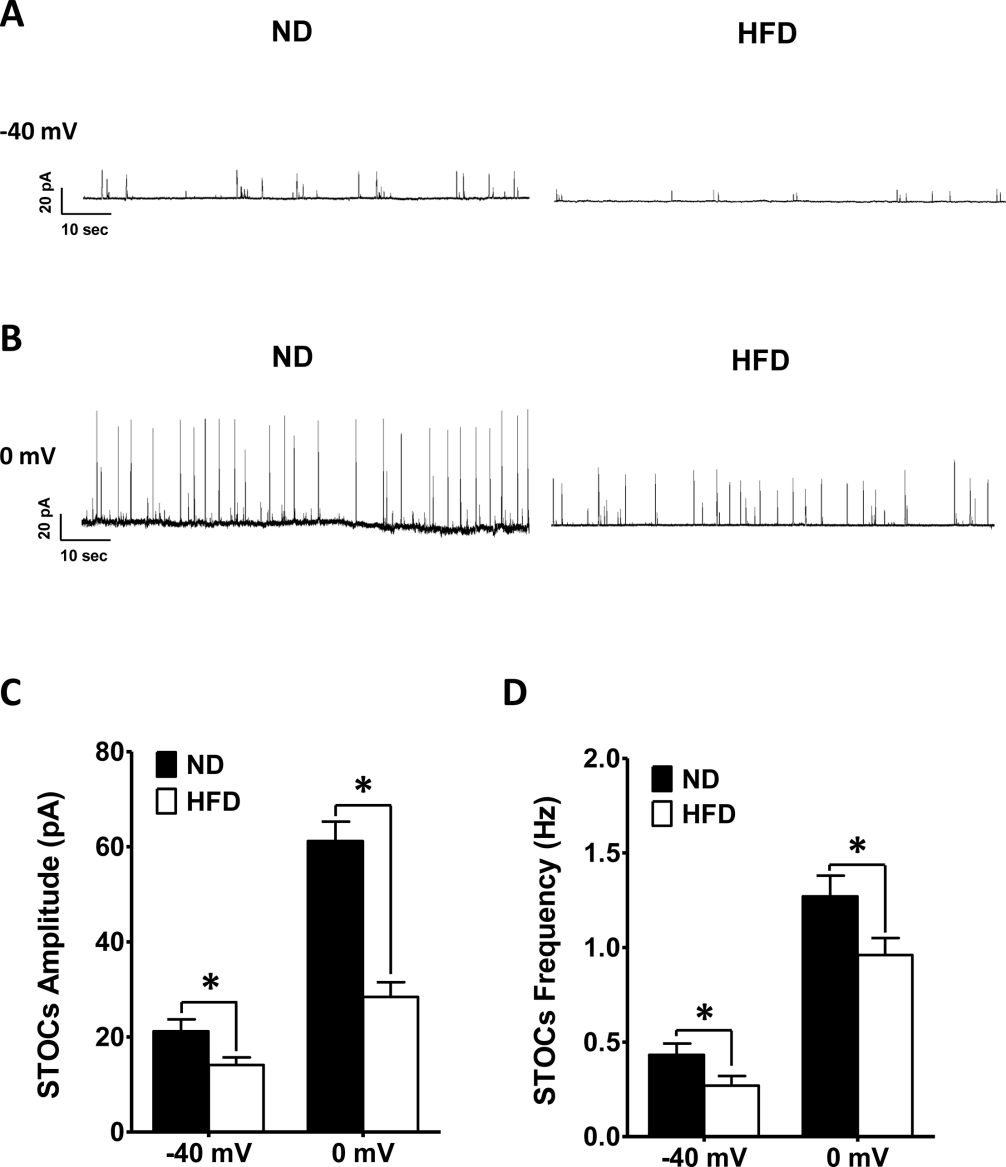
**The amplitude and frequency of STOCs are lower in HFD DSM cells than in ND DSM cells.** Representative STOC recordings in ND and HFD DSM cells at −40 mV. (**A**) and 0 mV (**B**). (**C**) The differences in the mean STOC amplitudes of ND and HFD DSM cells at −40 mV and 0 mV. (**D**) Comparison of the average STOC frequency in ND and HFD DSM cells at −40 mV and 0 mV. Data are expressed as the mean ± SEM; n = 14, N = 7 in the ND group, n = 14, N = 6 in the HFD group; * P < 0.05 for ND vs. HFD. ND: normal diet; HFD: high-fat diet; STOC: spontaneous transient outward current; *NS*: not significant.

At a holding potential of −40 mV, the amplitude of the STOCs was notably smaller in HFD DSM cells (14.12 ± 1.57 pA; n = 14, N = 6) than in ND DSM cells (21.17 ± 2.47 pA; n = 14, N = 7; P < 0.05; Figure 4A, 4C), and the frequency of the STOCs differed significantly between ND DSM cells (0.43 ± 0.06 Hz; n = 14, N = 7) and HFD DSM cells (0.27 ± 0.05Hz; n = 14, N = 6; P < 0.05; Figure 4A, 4D). In addition, at voltages higher than 0 mV, the STOCs of HFD DSM cells exhibited significantly lower amplitudes (HFD 28.41 ± 3.08 pA, n = 14, N = 6; ND 61.17 ± 4.12 pA, n = 14, N = 7; P < 0.05; Figure 4B, 4C) and frequencies (HFD 0.96 ± 0.09 Hz, n = 14, N = 6; ND 1.27 ± 0.11 Hz, n = 14, N = 7; P < 0.05; Figure 4B, 4D) than those of ND DSM cells.

**Figure 5:**
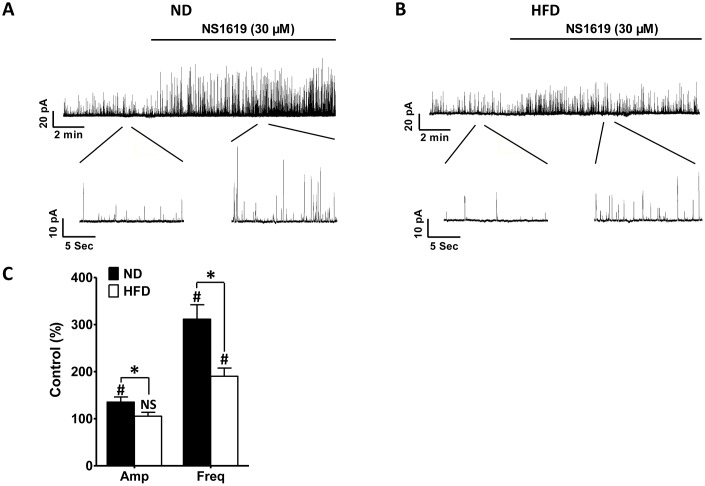
**NS1619-induced STOCs are significantly attenuated in HFD DSM cells.** Original recordings illustrating the effects of 30 μM NS1619 on STOCs in ND. (**A**) and HFD (**B**) DSM cells. (**C**) Summary data illustrating the differences in STOCs in the presence and absence of 30 μM NS1619 in ND and HFD DSM cells. Data are expressed as the mean ± SEM; n = 7, N = 5 per group; * P < 0.05 for ND vs. HFD; # P < 0.05 for control vs. NS1619. ND: normal diet; HFD: high-fat diet; STOCs: spontaneous transient outward currents; NS: not significant; Amp: amplitude; Freq: frequency.

In another set of experiments, NS1619, a selective BK channel activator, was applied to stimulate STOC activity in HFD DSM cells at a holding potential of −40 mV, which is very close to the physiologic membrane potential of intact DSM cells [21, 31]. In HFD DSM cells, NS1619 (30 μM) increased the frequency of STOCs to 190.21 ± 17.42% of the control values (n = 7, N = 5; P < 0.05; Figure 5B, 5C) without significantly changing the average amplitude of the STOCs (n = 7, N = 5; P > 0.05; Figure 5B, 5C). However, in the ND group, both the amplitude and frequency of STOCs were significantly enhanced to 135.42 ± 10.91% and 311.47 ± 30.61% of control activity, respectively, after the application of NS1619 (30 μM) (n = 7, N = 5; P < 0.05; Figure 5A, 5C). A weaker activating effect of NS1619 (30 μM) on STOCs was detected in HFD DSM cells than in ND DSM cells (P < 0.05; Figure 5C). Thus, DSM cells from HFD rats presented with weaker STOC activity than those from ND animals.

### NS1619-sensitive whole-cell BK currents are reduced in DSM cells from HFD rats

**Figure 6:**
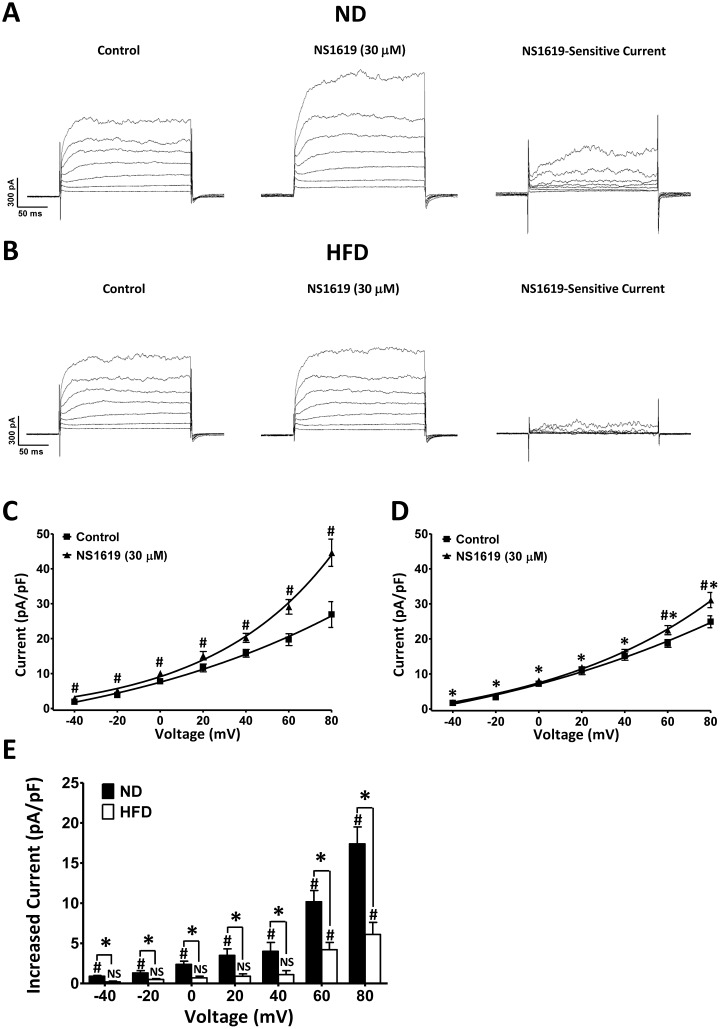
**NS1619-sensitive whole-cell BK currents are significantly lower in HFD DSM cells than in ND DSM cells.** Original recording illustrating the activating effect of 30 μM NS1619 on voltage-dependent whole-cell currents in ND. (**A**) and HFD (**B**) DSM cells. Current-voltage relationships illustrate the differences in whole-cell BK current density in the presence and absence of 30 μM NS1619 in ND (**C**) and HFD (**D**) DSM cells. (**E**) Summary data illustrating that 30 μM NS1619 had a significantly lower effect on whole-cell BK currents in HFD DSM cells than in ND DSM cells. Data are expressed as the mean ± SEM; n = 12, N = 7 in the ND group, n = 12, N = 8 in the HFD group; * P < 0.05 for ND vs. HFD; # P < 0.05 for control vs. NS1619. ND: normal diet; HFD: high-fat diet; *NS*: not significant.

It was previously demonstrated that an NS1619-sensitive whole-cell current occurs due to the activation of BK channels in DSM cells [15]. We applied a depolarizing voltage-step protocol at a holding potential of −70 mV to determine whether the NS1619-sensitive whole-cell BK current was altered in HFD animals. The average DSM cell capacitance did not differ significantly between the ND (26.31 ± 1.22 pF; n = 12, N = 7) and HFD (26.87 ± 1.38 pF; n = 12, N = 8; P > 0.05) groups. The current-voltage relationships illustrated that NS1619 significantly increased the whole-cell BK currents of DSM cells from both ND rats (n = 12, N = 7; P < 0.05; Figure 6A, 6C) and HFD rats (n = 12, N = 8; P < 0.05; Figure 6B, 6D). However, the NS1619-sensitive whole-cell BK currents were lower in HFD DSM cells than in ND DSM cells (P < 0.05; Figure 6E). Our voltage-clamp data indicated that the ability of BK channels to suppress DSM cell excitability was reduced in HFD rats.

### Hyperpolarization of the membrane potentialinduced by pharmacological activation of BK channels is attenuatedin DSM cells fromHFD rats

**Figure 7:**
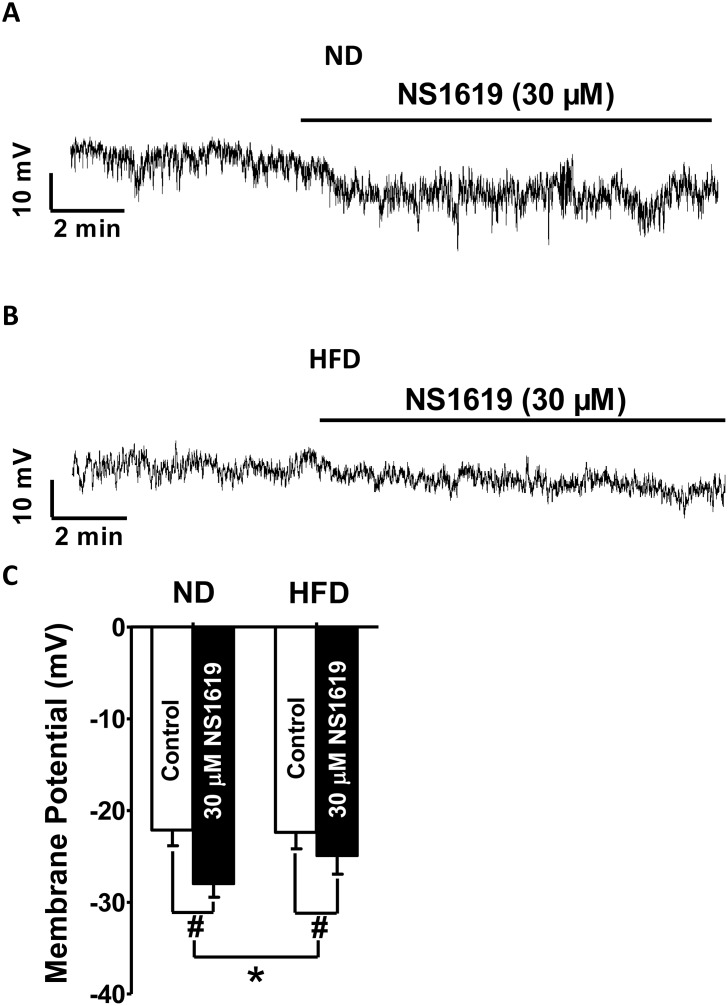
**Hyperpolarization of the membrane potential upon pharmacological activation of BK channels is attenuated in HFD DSM cells.** Representative recordings in current-clamp mode illustrating the NS1619 (30 μM)-induced hyperpolarization effect on the membrane potential in ND (**A**) and HFD (**B**) DSM cells. (**C**) Summary data indicating a significantly attenuated hyperpolarization effect on the membrane potential in HFD DSM cells compared with ND DSM cells. Data are expressed as the mean ± SEM; n = 8, N = 6 per group; * P < 0.05 for ND vs. HFD; # P < 0.05 for control vs. NS1619. ND: normal diet; HFD: high-fat diet; *NS*: not significant.

We next investigated whether HFD feeding for 12 weeks altered the ability of BK channels to hyperpolarize the membrane potential of DSM cells. The average DSM cell capacitance did not differ significantly between the ND (26.12 ± 1.09 pF; n = 8, N = 6) and HFD (27.17 ± 1.28 pF; n = 8, N = 6; P > 0.05) groups. In the absence of NS1619, the membrane potential did not differ significantly between the ND (n = 8, N = 6) and HFD (n = 8, N = 6; P > 0.05) groups. NS1619 (30 μM) significantly hyperpolarized the membrane potential from −22.12 ± 1.71 to −28.09 ± 1.41 mV in DSM cells from ND rats (n = 8, N = 6; P < 0.05; Figure 7A, 7C), and from −22.42 ± 1.81 to −24.91 ± 1.89 mV in DSM cells from HFD animals (n = 8, N = 6; P < 0.05; Figure 7B, 7C). However, the NS1619-induced hyperpolarization effect was smaller in DSM cells from HFD rats (2.82 ± 0.51 mV; n = 8, N = 6) than in DSM cells from ND rats (5.81 ± 0.69 mV; n = 8, N = 6; P < 0.05; Figure 7A–7C). Our current-clamp data established that HFD feeding attenuated the ability of BK channel activation to hyperpolarize the membrane potential of DSM cells.

### Selective pharmacological activation of BK channels with NS1619 attenuates spontaneous phasic contractions more weakly in isolated DSM strips from HFD rats

**Figure 8:**
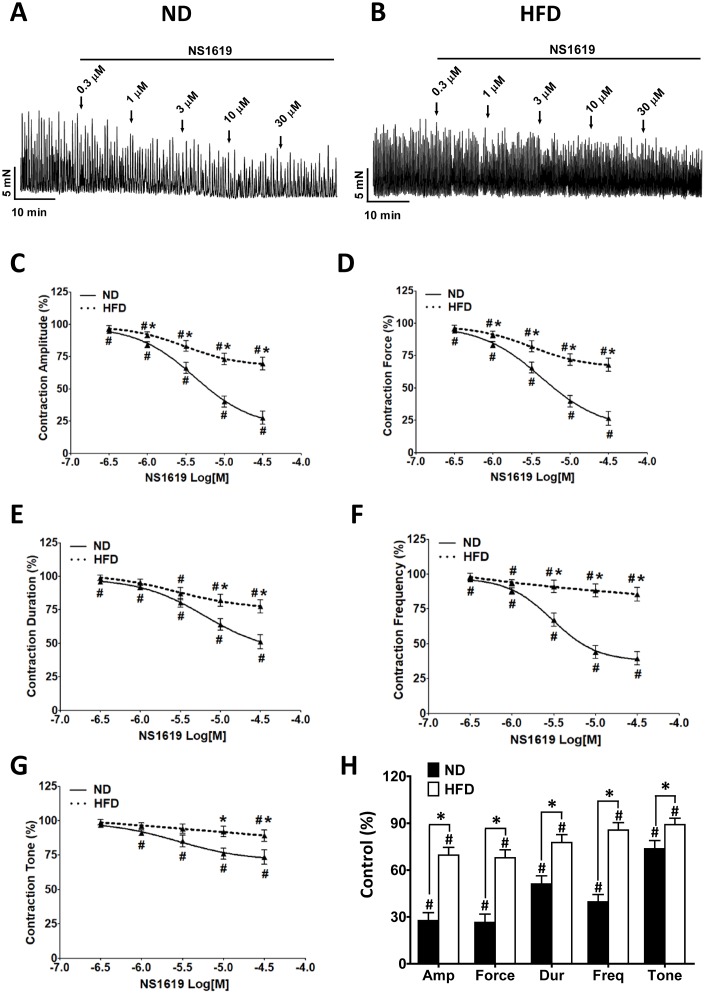
**The inhibitory effects of NS1619 on spontaneous phasic contractions are attenuated in DSM strips from HFD rats.** Representative recordings of DSM strips isolated from ND (**A**) and HFD (**B**) rats, illustrating the concentration-dependent inhibitory effects of NS1619 (0.3-30 μM) on spontaneous phasic contractions. Cumulative concentration-response curves illustrate the effects of NS1619 on the amplitude (**C**), muscle integral force (**D**), duration (**E**), frequency (**F**) and tone (**G**) of spontaneous phasic contractions in ND and HFD DSM strips. (**H**) Summary data illustrating that the inhibitory effects of 30 μM NS1619 on spontaneous phasic contractions in isolated DSM strips were lower in HFD rats than in ND rats. Data are expressed as the mean ± SEM; n = 11, N = 8 in the ND group, n = 11, N = 7 in the HFD group; * P < 0.05 for ND vs. HFD; # P < 0.05 for control vs. NS1619. ND: normal diet; HFD: high-fat diet; *NS*: not significant; Amp: amplitude; Dur: duration; Freq: frequency.

Our previous studies indicated that changes in DSM cell excitability can alter DSM contractility [13, 14]. In the present study, the pharmacological activation of BK channels with NS1619 (0.3-30 μM) dose-dependently relaxed the spontaneous phasic contractions in both groups (HFD n = 11, N = 7, P < 0.05; ND n = 11, N = 8, P < 0.05; Figure 8A–8G). However, consistent with the patch-clamp data, the inhibitory effect of NS1619 on the contractility of DSM strips was lower in HFD rats than in ND rats (Figure 8A–8H). At the highest concentration of NS1619 applied (30 μM), the amplitude was reduced to 27.61 ± 5.09% vs. 69.62 ± 4.91% of the control value (ND vs. HFD), the muscle integral force was reduced to 26.49 ± 5.29% vs. 67.91 ± 5.12%, the duration was reduced to 51.12 ± 5.21% vs. 77.62 ± 4.89%, the frequency was reduced to 39.63 ± 4.71%vs. 85.62 ± 4.84% and the tone was reduced to 73.62 ± 5.31%vs. 89.12 ± 4.09% (ND vs. HFD, respectively; P < 0.05; Figure 8H).

### The inhibitory effect of selective BK channel activation with NS1619 on electrical field stimulation-induced contractions is attenuated in DSM strips isolated from HFD rats

**Figure 9:**
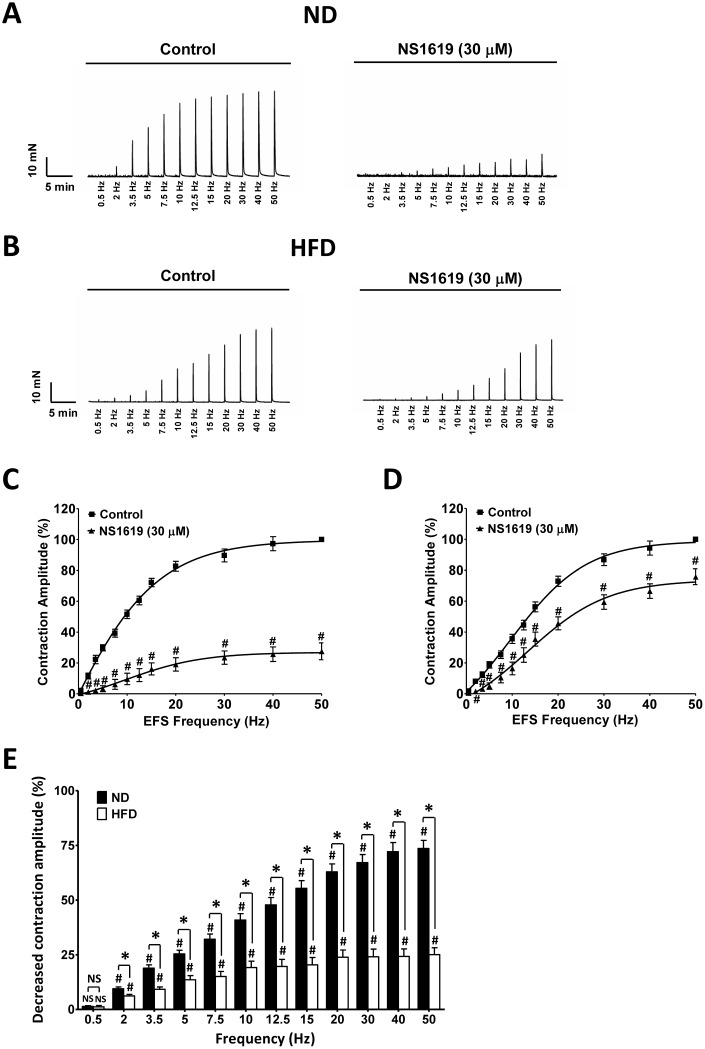
**The inhibitory effects of NS1619 on EFS-induced contractions are attenuated in HFD DSM strips.** Original recordings of DSM strips demonstrating the inhibitory effects of NS1619 (30 μM) on EFS-induced contractions (stimulation frequency 0.5-50 Hz) in the ND (**A**) and HFD (**B**) groups. The frequency response curves illustrate the differences in amplitude of EFS-induced contractions in the presence and absence of 30 μM NS1619 in ND (**C**) and HFD (**D**) DSM strips.(**E**) Summary data illustrating the inhibitory effects of 30 μM NS1619 on EFS-induced contractions at the highest stimulation frequency (50 Hz) in isolated DSM strips from both groups. Data are expressed as the mean ± SEM; n = 12, N = 7 in the ND group, n = 13, N = 7 in the HFD group; * P < 0.05 for ND vs. HFD; # P < 0.05 for control vs. NS1619. ND: normal diet; HFD: high-fat diet; *NS*: not significant.

Next, we evaluated the effects of NS1619 (30 μM) on electrical field stimulation (EFS)-induced contractions (stimulation frequency 0.5–50 Hz) in DSM strips from the two groups of rats. NS1619 significantly inhibited EFS-induced contractions in DSM strips from both ND (n = 12, N = 7; P < 0.05; Figure 9A, 9C) and HFD (n = 13, N = 7; P < 0.05; Figure 9B, 9D) rats. However, at most EFS frequencies, the relaxation effect of NS1619 on the amplitude of EFS-induced contractions was lower in DSM strips from HFD rats than in those from ND rats. At the highest EFS frequency of 50 Hz, NS1619 reduced the amplitude to 75.82 ± 5.13% of the control value in DSM strips isolated from HFD rats, but to 27.49 ± 5.51% of the control value in DSM strips isolated from ND animals (P < 0.05; Figure 9E).

In conclusion, this functional study established that the inhibitory effects of BK channels on DSM strip contractility were lower in HFD rats than in ND rats.

## DISCUSSION

The relationship between diet-induced obesity and OAB, which is supported by epidemiological studies, has become an important topic because of the high prevalence of these disorders in aging societies [8, 9]. HFD-fed animals are among the most commonly used models to investigate the etiology of OAB. Mice fed a HFD for 8 months were reported to have voiding dysfunction and lower-urinary-tract fibrosis [32]. OAB associated with an increase in purinoceptors was observed in rats fed a HFD for 24 weeks [33]. We previously observed OAB and increased inflammatory responses in the DSM in rats fed a HFD for 12 weeks [10, 34]. Notably, in many studies, including the present study, obese rats have been found to exhibit increased bodyweights without significant changes in bladder weight [10, 32, 33].

It is well known that obesity is strongly associated with OAB in rats, as evidenced by more frequent non-voiding contractions, shorter voiding intervals, a lower bladder capacity and a smaller voiding volume [10, 34, 35]. OAB is more of a storage issue than a voiding issue, so the maximum pressure during voiding has not been reported to change significantly in obese rats [10, 34, 35]. Obesity markedly increases the risk of benign prostatic hyperplasia, which thus increases the risk of OAB caused by partial bladder outlet obstruction (PBOO) [36, 37]. A high incidence of prostate enlargement was also discovered in HFD-induced hyperlipidemic rats [33]. In the present study, the use of female rats with HFD-induced obesity avoided the effects of obesity-associated benign prostatic hyperplasia and PBOO on bladder function. Therefore, our model of HFD-induced obesity, in which the rats displayed increased bodyweights and urodynamically established OAB, closely mirrored the changes reported in humans.

The BKα, BKβ1 and BKβ4 subunits have been detected in the DSM at both the mRNA and protein levels [15, 18–21, 23, 24]. Here, qRT-PCR was performed on whole DSM tissues as well as freshly isolated DSM cells to eliminate the effects of other cell types in the DSM layer on the detection of the BK channel subunits [14, 23]. The BKα subunit forms the functional pore of the BK channel, whereas the BKβ1 subunit performs the regulatory functions of enhancing BK channel Ca^2+^ sensitivity and modifying BK current activity [18, 19, 25, 38]. Therefore, changes in the expression of either of these subunits could profoundly alter DSM excitability and contractility. The absence of the BKα subunit was found to cause significant depolarization of the DSM cell membrane potential, along with increased DSM contractility and urodynamically demonstrable OAB [25, 28, 39]. In addition, the deletion of the smooth muscle-specific *Kcnmb1* was reported to enhance DSM contractility markedly in mice, indicating that the BKβ1 subunit constitutively regulates DSM function [38]. BK channel subunit expression was shown to be significantly reduced in the DSM of patients with OAB resulting from benign prostatic hyperplasia and neurogenic bladder dysfunction [27, 28]. We found that the mRNA levels of the *Kcnma1 and Kcnmb1* were significantly reduced in the DSM of HFD rats, consistent with previous studies. These studies provide direct molecular evidence that attenuated expression of the *Kcnma1 and Kcnmb1* promotes HFD-induced OAB.

The activation of the BK channels induces STOCs and steady-state whole-cell BK currents to hyperpolarize the membrane potential and subsequently reduce DSM excitability [15, 16, 20, 21]. The activation of STOCs and steady-state whole-cell BK currents was found to be significantly attenuated both in artery myocytes from hypoxic rats and in DSM cells from neurogenic OAB patients [27, 40]. In DSM cells from rats with detrusor overactivity secondary to PBOO, the ability of BK channels to hyperpolarize the membrane potential was shown to be markedly reduced [41]. Here, we investigated whether HFD feeding reduced BK channel function in DSM cells by performing the perforated patch-clamp technique, which directly assesses ion channel activity while preserving intracellular signaling mechanisms. The amplitude and frequency of STOCs were significantly lower in HFD DSM cells than in ND DSM cells (Figure 4). This attenuation of STOCs may have enhanced DSM cell excitability.

NS1619, a selective BK channel opener, significantly activated STOCs, steady-state whole-cell BK currents and membrane potential hyperpolarization in DSM cells. Our voltage-clamp data indicated that the activating effects of NS1619 on STOCs and steady-state whole-cell BK currents were dramatically attenuated in DSM cells from HFD rats (Figures 5, 6). Moreover, according to our current-clamp data, the hyperpolarization effect of NS1619 on the DSM membrane potential was significantly reduced in HFD rats (Figure 7). Therefore, the reduced inhibitory effects of BK channels on DSM cell excitability resulted in OAB in the HFD rats.

BK channels are also fundamental inhibitors of DSM contractility [15, 16, 18–21, 27]. DSM spontaneous phasic contractions are associated with spontaneous action potentials, and reflect the transient elevation of the intracellular Ca^2+^ concentration through L-type voltage-dependent Ca^2+^channels [18, 19, 21, 42]. Therefore, spontaneous phasic contractions are the most important characteristic of the DSM, and are associated with detrusor overactivity and OAB. BK channels restore the membrane potential after the initial depolarization phase of the action potential caused by L-type voltage-dependent Ca^2+^ channels, and thereby inhibit spontaneous phasic contractions in the DSM [17, 43]. In the present study, the inhibitory effects of NS1619 on every parameter of spontaneous DSM contractility were significantly attenuated in the HFD group (Figure 8). Our results are in agreement with recent reports describing the reduced effects of NS1619 or iberiotoxin, a BK channel inhibitor, on spontaneous DSM contractions in neurogenic OAB patients [27, 44].

DSM contractions during voiding result from the activation of purinergic P2X receptors and muscarinic receptors by ATP and acetylcholine, which are released by parasympathetic nerves. EFS-induced contractions can activate the cholinergic and purinergic nerves located in isolated DSM strips. The inhibitory effect of NS1619 on the amplitude of EFS-induced contractions was significantly lower in DSM strips from HFD rats than in those from ND rats (Figure 9). Consistent with the patch-clamp and molecular data, the results of our functional studies established that reduced BK channel activity increases DSM contractility in HFD rats.

The complicated etiology of OAB is not completely understood [1]. It is well known that OAB, which is generally induced by PBOO, obesity and neurogenic disorders, is associated with increased DSM excitability and contractility [1, 13, 14, 20, 21, 27, 41]. The expression and activity of BK channels, the major inhibitors of DSM excitability and contractility, have been reported to be reduced in the DSM of patients with OAB resulting from benign prostatic hyperplasia and neurogenic bladder dysfunction [27, 28]. The present study provides strong evidence that attenuated BK channel expression and activity are also associated with OAB resulting from HFD-induced obesity. Thus, although the etiologies of PBOO-, neurogenic disorder- and obesity-induced OAB are different, similar reductions in the expression and activity of BK channels have been discovered in the DSM [27, 28, 41]. One explanation for these similar phenomena is that, regardless of the etiology of OAB, similar compensatory mechanisms reduce the expression and activity of BK channels and thus increase the excitability and contractility of the DSM.

Recently, sacral neuromodulation is widely reported, however it is only the second-line treatment of refractory OAB [45]. The first-line treatment of OAB is still pharmacotherapy [45–47]. Acetylcholine is the main neurotransmitter in the DSM. Antimuscarinic agents can competitively inhibit acetylcholine activity in DSM cells and on bladder wall receptors, thereby reducing afferent signaling and blocking DSM contractions; thus, such agents are the first-line drug therapy for OAB [46, 47]. However, conventional antimuscarinic pharmacotherapies cause side effects such as dry mouth, constipation and headaches especially in older adults, which limit the application of these drugs [48]. Recently, NS1619, a selective BK channel opener, was found to inhibit human DSM excitability and contractility significantly [16]. However, the currently available selective BK channel openers are not selective for DSM tissue, and thus have collateral effects elsewhere in the body. In addition, potassium channels (including BK channels) have been suggested as treatment targets for OAB in numerous studies, but all the potassium channel activators tested in humans have been unsuccessful. A genetic approach to enhancing BK channel expression has also emerged as a successful therapeutic strategy. OAB caused by PBOO in rats was eliminated by the injection of naked DNA encoding BK channels [49]. Therefore, the selective activation of BK channel subunits could be used to treat OAB without significantly impacting other organs.

## CONCLUSIONS

In conclusion, this study has demonstrated the function of BK channels in OAB. We successfully generated a rat model of OAB resulting from HFD-induced obesity. Reduced mRNA levels of the pore-forming *Kcnma1* and the regulatory *Kcnmb1* were detected in the DSM of the HFD rats. Perforated patch-clamp experiments strongly established that BK channel activity was attenuated in these rats. In DSM cells from HFD rats, STOC activity and NS1619-sensitive whole-cell BK currents were reduced, and the ability of BK channels to hyperpolarize the membrane potential was weakened. Functional studies revealed that the inhibitory effects of NS1619 on spontaneous and EFS-induced contractions were attenuated in HFD DSM strips. These findings indicate that reduced BK channel expression and activity are associated with HFD-induced detrusor overactivity, and that activating particular BK channel subunits could be an effective therapeutic strategy for OAB.

## MATERIALS AND METHODS

### Animals

In total, 80 seven-week-old female Sprague-Dawley rats (China Medical University, Shenyang, Liaoning, China) with an average weight of 204.72 ± 7.21 g were used in this study. For 12 weeks, the study animals were housed three per cage on a 12-h light-dark cycle, and were fed either the ND (fat: 5%; protein: 20%; carbohydrate: 75%) or an obesity-inducing HFD (fat: 30%; protein: 14%; carbohydrate: 56%), as described previously [10, 11] (N = 40 per group). All rats were weighed at 12 weeks, and urodynamic studies were conducted in 10 rats from each group. The study animals were then killed in a CO_2_ tank, and bladder specimens were collected. All experimental procedures were approved by the Institutional Animal Care and Use Committee of China Medical University.

### Cystometry

General anesthesia was induced by 5% isoflurane/O_2_ gas inhalation through a facial mask. After the bladder was surgically exposed, a catheter was inserted into the dome of the bladder and connected to a physiological pressure transducer and an injection pump (DantecMenuet; DantecMedical A/S, Skovlunde, Denmark). Cystometry was performed by the infusion of warm saline (37-38°C) into the bladder at a rate of 12 mL/h. Three voiding events were recorded for each rat, and the following parameters were assessed: maximum pressure during voiding (maximal pressure developed during a voiding cycle), bladder capacity (volume of saline infused to induce voiding), voiding volume (micturition volume), voiding interval (interval between voids) and number of non-voiding contractions during one voiding event. Non-voiding contractions were defined as spontaneous contractions (>4 cmH_2_O from the baseline bladder pressure) that did not result in voiding. Bladders that were assessed by cystometry were not used in other experiments (N = 10 per group).

### DSM tissue collection

The rats were euthanized by CO_2_ inhalation, and thoracotomy was performed. The urinary bladders were removed rapidly and preserved in a cold dissection solution. The urinary bladders were then cut open longitudinally, and the mucosa were removed. DSM strips (5–7 mm long and 2– 3 mm wide) were collected from the dome of the urinary bladder.

### DSM single-cell isolation

Single DSM cells were freshly isolated as described previously [14, 20, 21]. Briefly, one to two DSM strips were incubated in 2 mL of dissection solution supplemented with 1 mg/mL bovine serum albumin (BSA), 1 mg/mL papain and 1 mg/mL DL-dithiothreitol at 37°C for 12–18 min. The DSM strips were transferred to 2 mL of dissection solution supplemented with 1 mg/mL BSA, 0.5 mg/mL type II collagenase, 0.5 mg/mL trypsin inhibitor and 100 μM CaCl_2_, and were incubated at 37 °C for 12–15 min.

### qRT-PCR

The TRIzol reagent (Invitrogen, Waltham, MA, USA) was used to isolate total RNA from mucosa-free rat DSM strips and enzymatically isolated DSM cells (n = 16, N = 5 per group). For single cell qRT-PCR, n = the number of strips to isolate cells, and N = the number of rats. Total RNA was reverse transcribed with the SuperScript^TM^ First-Strand Synthesis System (Invitrogen) according to the manufacturer’s instructions. Real-time PCR was then performed with the synthesized cDNA on an ABI Prism7500 sequence detection system with SYBR GreenPCR Master Mix (ThermoFisher Scientific, Fair Lawn, NJ, USA). Real-time PCR was carried out to analyze the mRNA expression of the *Kcnma1, Kcnmb1* and *Kcnmb4* and *Gapdh* with specific primers (Table 1). The PCR conditions were 94°C for 1 min, followed by 95°C for 30 s and 58°C for 40 s, for a total of 35 cycles. All reactions were performed three times and normalized to *Gapdh*. All qRT-PCR products from intact whole DSM tissues and isolated DSM cells were purified with a GenElute PCR Clean-Up Kit (Sigma-Aldrich, St.Louis, MO, USA), and the sequences of the detected genes were confirmed by direct sequencing of the amplified PCR products.

**Table 1:** Primers used for the qRT-PCR experiments.

**Gene**	**Forward**	**Reverse**	**GenBank no.**	**Size (bp)**
*Kcnma1*	AGGCTCTGTTCAAACGGCAT	TTGAGCAGATGGGCCTTGTT	NM_031828	250
*Kcnmb1*	ACCCATGCCTTTGGGTCAAT	ATAGAGGCGCTGGTACACAA	NM_019273	236
*Kcnmb4*	TCAACATCAAAGACCAGAGGAC	TGAGGACACCCACCACAAAC	NM_023960	107
*Gapdh*	GTTACCAGGGCTGCCTTCTC	ACCAGCTTCCCATTCTCAGC	XM_001067272.2	152

bp, base pairs.

### Electrophysiological recordings

The amphotericin B-perforated whole-cell patch-clamp technique was performed to record the membrane potentials and whole-cell currents of freshly isolated single rat DSM cells, as described previously [14, 20, 21]. Patch-clamp recordings were made on an Axopatch 200B amplifier system and a Digidata 1440A controlled with pCLAMP 10.2 software (Molecular Devices, Union City, CA, USA). The recording currents were filtered at 1 kHz with an eight-pole Bessel filter (model 900CT/9L8L; Frequency Devices, Ottawa, IL, USA) and sampled at a rate of 10 kHz. The pipettes, prepared from borosilicate glass, were pulled and polished to achieve a final tip resistance of 4–7 MΩ. The DSM cell membrane potential was recorded in the current-clamp mode of the patch-clamp technique without any current input (Ih = 0; n = 8, N = 6 per group). STOCs were recorded at a holding potential of −40 to 0 mV in voltage-clamp mode (n = 21, N = 12 in the ND group and n = 21, N = 11 in the HFD group). For the recording of whole-cell K^+^ currents, DSM cells were held at −70 mV; then, voltage depolarization was performed from −40 to +80 mV for 200 ms in 20-mV steps, and the cells were then repolarized to −70mV (n = 12, N = 7 in the ND group and n = 12, N = 8 in the HFD group). All patch-clamp experiments were conducted at room temperature (22–23°C).

### Isometric DSM tension recordings

Isometric DSM contraction recordings were performed as described previously [13, 20, 21]. Isolated DSM strips were secured to isometric force-displacement transducers and placed in a physiological saline solution aerated with 95% O_2_/5% CO_2_, pH 7.4, at 37°C. The DSM strips were initially tensioned (10 mN) during an equilibration period of 45–60 min. The effects of neurotransmitters released from neurons in the DSM were minimized through the use of 1 μM tetrodotoxin, a selective inhibitor of neuronal voltage-gated Na^+^ channels, during the recording of spontaneous phasic contractions (n = 11, N = 8 in the ND group and n = 11, N = 7 in the HFD group).

In another experimental series, nerve-evoked contractions were induced by EFS with a pair of platinum electrodes mounted in a tissue bath parallel to a DSM strip in the absence of tetrodotoxin. The EFS pulses were generated with a PHM-152I stimulator (MED Associates, St.Albans, VT, USA). The EFS pulse parameters were as follows: 0.75 ms pulse width, 20 V pulse amplitude, 3 s stimulus duration, and polarity reversal for alternating pulses. After an equilibration period, the DSM strips were subjected to continuous repetitive EFS at increasing frequencies from 0.5–50 Hz at 3-min intervals. Contractions were recorded (n = 12, N = 7 in the ND group and n = 13, N = 7 in the HFD group) with a MYOMED myograph system (MED Associates).

### Solutions and drugs

The dissection solution contained the following components: 80 mM monosodium glutamate, 55 mM NaCl, 6 mM KCl, 10 mM glucose, 10 mM HEPES and 2 mM MgCl_2_; the pH was adjusted to 7.3 with NaOH. The extracellular solution for the patch-clamp experiments contained the following components: 134 mM NaCl, 6 mM KCl, 1 mM MgCl_2_, 2 mM CaCl_2_, 10 mM glucose and 10 mM HEPES; the pH was adjusted to 7.4 with NaOH. The pipette solution contained the following components: 110 mM potassium aspartate, 30 mM KCl, 10 mM NaCl, 1 mM MgCl_2_, 10 mM HEPES and 0.05 mM EGTA; the pH was adjusted to 7.2 with NaOH, and the solution was supplemented with 200 μg/mL amphotericinB freshly dissolved in dimethyl sulfoxide (DMSO). The physiological saline solution was freshly prepared daily and contained the following components: 119 mM NaCl, 4.7 mM KCl, 24 mM NaHCO_3_, 1.2 mM KH_2_PO_4_, 2.5 mM CaCl_2_, 1.2 mM MgSO_4_ and 11 mM glucose; the solution was aerated with 95% O_2_/5% CO_2_ to obtain a pH of 7.4. The trypsin inhibitor, BSA and amphotericin B were obtained from ThermoFisher Scientific. Papain was purchased from Worthington Biochemical (Lakewood, NJ, USA). NS1619 (1-(2′-hydroxy-5′-trifluoromethylphenyl)-5-trifluoromethyl-2 (3H)benzimidazolone) was purchased from Sigma-Aldrich. Amphotericin B and NS1619 were dissolved in DMSO, while all other chemicals were dissolved in double-distilled water. The maximum DMSO concentration in the bath solution did not exceed 0.1%.

### Data analysis and statistics

Relative differences in mRNA levels were calculated by the comparative Ctmethod (2^−ΔΔCt^) after the Ct values of the reference (*Gapdh*) and target (*Kcnma1, Kcnmb1* or *Kcnmb4*) genes were determined in each sample [10, 13, 14]. The relative mRNA level of the target gene was calculated after normalization to *Gapdh* expression.

The membrane potential was measured as the average of the last 5 min of recording under each experimental condition, and was analyzed with Clampfit 10.2 (Molecular Devices). The amplitude and frequency of STOCs, analyzed with MiniAnalysis software (Synaptosoft, Decatur, GA, USA), were normalized to their controls from the same cell. The mean value of the last 50-ms pulse of the 200-ms depolarization step of the recording was analyzed with Clampfit 10.2 and used to plot the current-voltage relationship. MiniAnalysis software (Synaptosoft) was applied to analyze five parameters of DSM contractions: the contraction amplitude, muscle integral force (area under the curve of the phasic contractions), duration (defined as the width of the contraction at 50% of the amplitude), frequency (contractions per minute) and tone (phasic contractions in the baseline curve). For the analysis of the effects of the compounds, one 5-min-long stable recording made prior to the application of the compounds was analyzed for the control, and another 5-min-longstable recording was analyzed after the application of each concentration of the compounds. For spontaneous phasic contractions, each parameter under the control conditions was taken to be 100%, and the data were normalized. The contraction amplitude at each EFS frequency was normalized to the amplitude at an EFS frequency of 50 Hz under the control conditions (taken to be 100%) and expressed as a percentage of the EFS-induced contraction.

The data were further analyzed with GraphPad Prism 5.0 software (GraphPad Software, San Diego, CA, USA). Data are expressed as the mean ± standard error of the mean (SEM); n = the number of strips or cells, and N = the number of rats. For single cell qRT-PCR, n = the number of strips to isolate cells, and N = the number of rats. Statistical significance was tested with one-way analysis of variance, followed by Dunnett’s multiple comparison test, Student’s t-test or paired t-test. P values < 0.05 were considered statistically significant.
